# Evaluating the relative importance of different blood pressure indices in screening for NAFLD: a survey report based on a health examination population

**DOI:** 10.3389/fcvm.2024.1338156

**Published:** 2024-04-29

**Authors:** Chong Hu, Ziqi Yu, Changli Wei, Guotai Sheng, Jianyong Chen, Yang Zou

**Affiliations:** ^1^ Department of Gastroenterology, Jiangxi Provincial People’s Hospital, The First Affiliated Hospital of Nanchang Medical College, Nanchang, Jiangxi, China; ^2^ Munich Medical Research School, LMU Munich, Munich, Germany; ^3^ Jiangxi Cardiovascular Research Institute, Jiangxi Provincial People’s Hospital, The First Affiliated Hospital of Nanchang Medical College, Nanchang, Jiangxi, China

**Keywords:** blood pressure indices, NAFLD, evaluating, non-alcoholic fatty liver disease, survey report

## Abstract

**Objective:**

While hypertension is a well-recognized risk factor for non-alcoholic fatty liver disease (NAFLD), the specific roles of various common blood pressure measurements [diastolic blood pressure (DBP), systolic blood pressure (SBP), pulse pressure (PP), mean arterial pressure (MAP)] in detecting NAFLD and evaluating the associated risk in adults remain unclear.

**Methods:**

A retrospective analysis was conducted on 14,251 adult participants undergoing health screenings in the NAfld in the Gifu Area, Longitudinal Analysis project (NAGALA). Following the Z-transformation of the independent variables, we evaluated the relationships between the four blood pressure indices and NAFLD through multivariable logistic regression models. This analysis documented the odds ratio (OR) and 95% confidence interval (CI) for each standard deviation (SD) increase. Additionally, the effectiveness of these indices in identifying NAFLD was comparatively analyzed using receiver operating characteristic (ROC) curves.

**Results:**

After adequately adjusting for confounders, all blood pressure indices except PP showed a positive correlation with NAFLD. For each SD increment, MAP had the strongest association with NAFLD compared to SBP and DBP. This finding was confirmed in populations without exercise habits, under 60 years of age, with normal blood pressure, and in non-obese groups. Furthermore, based on ROC analysis, MAP was found to have the highest accuracy in identifying NAFLD compared to the other three blood pressure indices.

**Conclusion:**

Among the four blood pressure indices evaluated, MAP demonstrates the greatest efficacy in identifying NAFLD and assessing its associated risk. These findings underscore the potential of MAP as the most promising blood pressure index for screening NAFLD.

## Introduction

NAFLD is a prevalent non-communicable disease, primarily characterized by fat accumulation and associated inflammation in the liver (1, 2). Recent global surveys reveal that approximately 30% (around 2.2 billion) of adults worldwide are affected by NAFLD (3), surpassing the total number of individuals with obesity (650 million) and diabetes (529 million) (4, 5). Despite the startling prevalence of NAFLD, the greater concern lies in its potential to cause chronic damage to the liver and extra-hepatic organ systems during its progression (1, 2, 6). Advanced stages of NAFLD can severely affect health and even be life-threatening, leading to a substantial disease burden (6–8). Considering NAFLD's high prevalence and escalating disease burden, alongside limited treatment options (9), prevention emerges as a crucial public health strategy. There is a pressing need for enhanced screening and assessment of modifiable risk factors for NAFLD.

Hypertension, with a global prevalence of 31.1% (10), is a key modifiable risk factor in NAFLD's development and progression (11, 12). Recent meta-analyses have shown that the presence of hypertension significantly increased the risk of NAFLD events by 47% (13), and the latest evidence from Mendelian randomization analysis based on Genome-Wide Association Studies further indicated a causal relationship between hypertension and commonly measured blood pressure parameters SBP, DBP, and NAFLD (14). Additionally, recent observational studies have confirmed that other blood pressure indices, such as PP and MAP, were also positively correlated with NAFLD, where elevated PP and MAP increased the risk of NAFLD (15, 16). In terms of NAFLD reversal, published studies have shown that controlling SBP/DBP below 140/90 mmHg in non-obese hypertensive patients was independently associated with a 40% reduction in NAFLD prevalence (17). Furthermore, clinical practice guidelines for NAFLD management by the European Association for the Study of the Liver/Diabetes/Obesity recommend close monitoring of NAFLD patients with hypertension, as the presence of hypertension leads to a higher risk of NAFLD disease progression (18). Given the current pandemic of hypertension and its significant impact on the development and progression of NAFLD, actively exploring the roles of various blood pressure indices in NAFLD screening are vital. However, there has been no systematic analysis of the different blood pressure indices in assessing or identifying the risk of NAFLD in adults. Therefore, to fill this gap in the field, our current study aimed to analyze and compare the relative importance of the four blood pressure indices SBP, DBP, PP, and MAP in identifying/assessing the risk of NAFLD.

## Methods

### Data source

In this study, we analyzed data from the NAGALA project dataset spanning from 1994 to 2016. This dataset, collated by Professor Okamura and colleagues, has been made publicly available in the Dryad database (19). In accordance with the Dryad database's terms of use and the Creative Commons Attribution-NonCommercial-ShareAlike 4.0 International License (CC BY-NC-ND 4.0), we utilized this dataset for secondary creation, duly crediting the source (19).

### Study design and population

The NAGALA project is a cross-sectional and longitudinal survey based on a health examination population, aimed at detecting common chronic diseases and their risk factors to promote public health. The detailed design and study outcomes have been published elsewhere (20). Briefly, the NAGALA project, initiated in 1994 and ongoing, recruited adults undergoing health check-ups at the Murakami Memorial Hospital in Gifu, Japan. The project was approved by the hospital's ethics committee, and informed consent for data use was obtained from each participant.

In their initial study, Okamura et al. included 20,944 participants from the NAGALA project (1994–2016), analyzing the role of ectopic fat obesity in diabetes onset. They excluded participants with (i) diagnosed diabetes, alcoholic fatty liver disease, viral hepatitis, or fasting plasma glucose (FPG) ≥6.1 mmol/L at baseline (*n* = 1,547); (ii) undergoing medication treatment at baseline (*n* = 2,321); (iii) excessive alcohol consumption (*n* = 739); (iv) missing baseline data (*n* = 863); (v) unexplained study withdrawal (*n* = 10), resulting in a final sample of 15,464 participants for their analysis.

**Figure 1:**
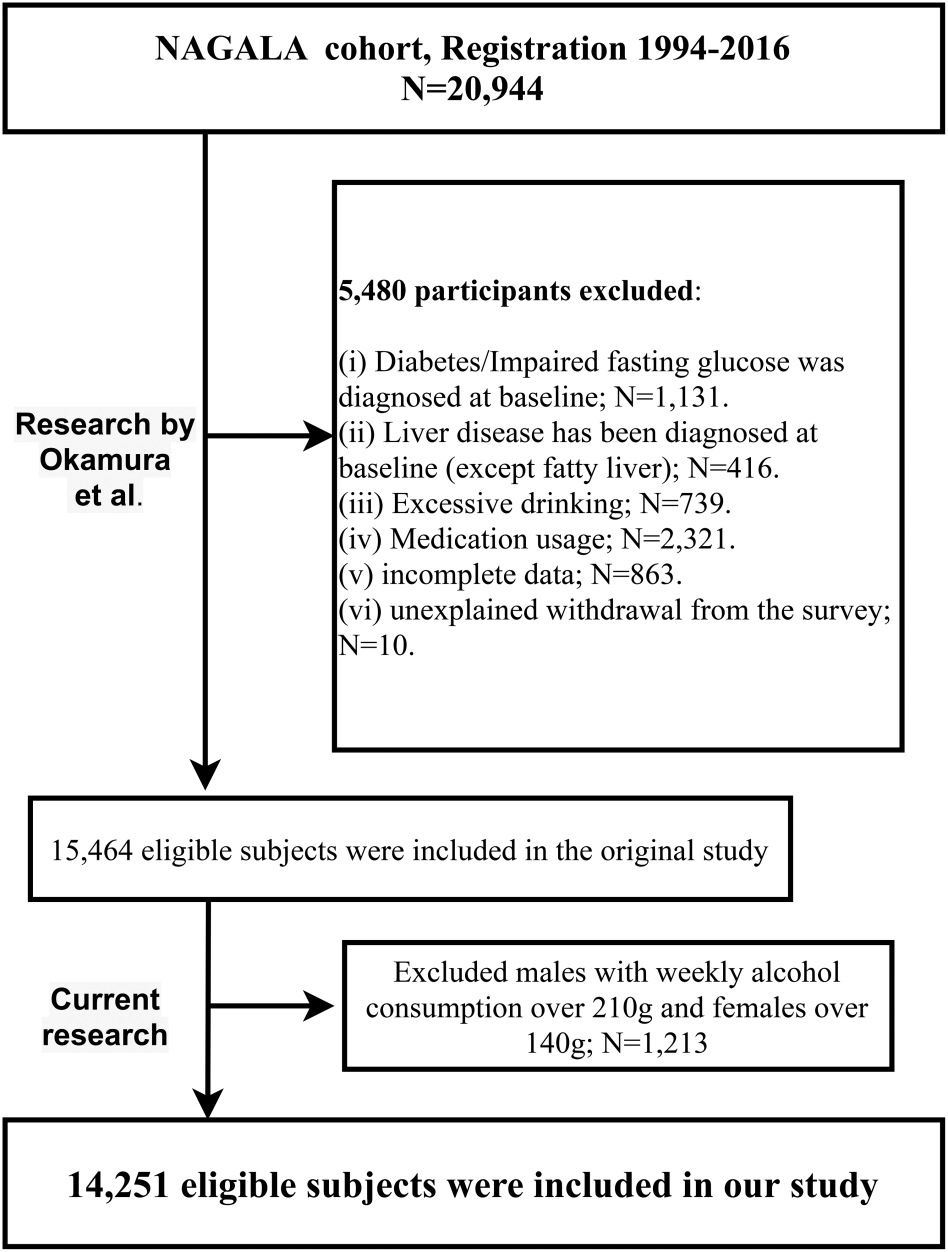
Flow chart for inclusion and exclusion of study participants.

Our current study, a secondary analysis of the NAGALA dataset, aimed to evaluate the relative importance of four blood pressure indices in assessing NAFLD. Building upon Okamura et al.'s research, we further excluded males with weekly alcohol consumption over 210 g and females over 140 g (*n* = 1,213), in accordance with the safe alcohol consumption limits of NAFLD (21). This resulted in 14,251 participants included in our analysis, as shown in the flowchart in Figure 1. As a secondary analysis, the Jiangxi Provincial People's Hospital ethics committee approved our study; the need for re-signing informed consent was waived due to prior agreements in the original research.

### Data collection

In the NAGALA project, all participants were required to fill out baseline questionnaires on demographic data (gender, age), lifestyle habits (smoking/drinking status, exercise habits), and chronic disease history (diabetes, liver diseases). Trained medical staff in a standardized environment measured simple physical parameters [including height, weight, waist circumference (WC)] and biochemical indices using automated biochemistry analyzers. Lifestyle habits were categorized as follows: (i) exercise habit defined as participating in physical activities at least once a week; (ii) drinking status based on weekly consumption in the past month, categorized as <40 g as none or minimal, 40–140 g as light, and 140–210 g as moderate (21, 22); (iii) smoking status defined based on smoking history as non-smoking, past smoking, and current smoking.

Blood pressure measurement is conducted in a quiet environment. After resting for 5 min, participants, assisted by medical personnel, supported both arms at heart level. The cuff is wrapped around the upper arm, adjusted to fit snugly, with the lower edge approximately half an inch above the elbow, and the air tube aligned with the midpoint of the participant's arm. Subsequently, SBP and DBP are recorded as the first and fifth Korotkoff sounds, respectively, using a mercury sphygmomanometer. The process is repeated three times, with a 2-min interval between measurements. The final recorded blood pressure value is the average of the second and third measurements.

Venous blood samples for biochemical analysis were taken after overnight fasting and analyzed in a standard laboratory for high-density lipoprotein cholesterol (HDL-C), total cholesterol (TC), triglycerides (TG), hemoglobin A1c (HbA1c), FPG, and concentrations of gamma-glutamyl transferase (GGT), alanine aminotransferase (ALT), aspartate aminotransferase (AST).

### Calculations

Body mass index (BMI) was calculated as weight in kilograms divided by height in meters squared;

PP was calculated as SBP minus DBP (23);

MAP was calculated as one-third of SBP plus two-thirds of DBP (23).

### NAFLD diagnosis

NAFLD was diagnosed through abdominal ultrasonography, after excluding other liver diseases and confirming alcohol consumption within safe limits for NAFLD (21). The ultrasound was performed by professional technicians, and gastroenterology experts, blind to the participants' details, diagnosed NAFLD based on criteria such as liver brightness, hepatorenal echo contrast, vascular blurring, and deep attenuation (24).

### Statistical analysis

Data in the current study were analyzed using Empower(R) Version 2.0 and R language 4.2.1. Baseline information was described as frequency (%) for categorical data and mean (SD) or median (interquartile range) for continuous data. Marginal structural models were employed to quantify differences in baseline characteristics of the study population (25, 26).

The association between the four blood pressure indices and NAFLD was analyzed using multivariable logistic regression models. Before inclusion in the regression models, the four indices were *Z*-transformed to eliminate disparities in OR due to different magnitudes of blood pressure values, ensuring comparability of the OR values calculated. Moreover, variance inflation factors were calculated to assess collinearity between the four indices and other covariates (Supplementary Tables S1–S4) (27); with a critical value of 5 for variance inflation factors, collinearity was found between the four indices and weight, WC. In models with NAFLD as the dependent variable, SBP and DBP showed collinearity with other blood pressure indices except themselves (Supplementary Tables S1 and S2); whereas, in models with PP and MAP as independent variables, no collinearity was found between PP and MAP, but they showed collinearity with SBP and DBP (Supplementary Tables S3 and S4). After identifying factors with multicollinearity, three progressively adjusted multivariable models were established. Model I primarily considered demographic data effects, adjusting for gender, age, height, and BMI. Model II further considered lifestyle factors, adjusting for smoking status, drinking status, and exercise habits. Model III, the final model, adjusted for all non-collinear variables, with mutual adjustment of PP and MAP in their respective models.

Several sensitivity analyses were conducted to verify the reliability of the association between the four blood pressure indices and NAFLD. (i) To mitigate the potential influence of exercise on NAFLD (28), we replicated the analysis based on Model III in the population without exercise habits. (ii) As aging is a significant contributor to NAFLD (29), and to reduce this influence, we also conducted the same analysis in participants younger than 60 years. (iii) The analysis was continued in the normotensive population. (iv) Since obesity is a major promoter of NAFLD (1, 2), we further validated the stability of the associations between the four indices and NAFLD in the non-obese population.

Using ROC analysis and Delong's test (30), we further evaluated and compared the ability of the four blood pressure indices to identify NAFLD, calculating the corresponding area under the curve (AUC), optimal threshold, sensitivity, and specificity.

## Results

### Baseline characteristics

**Figure 2:**
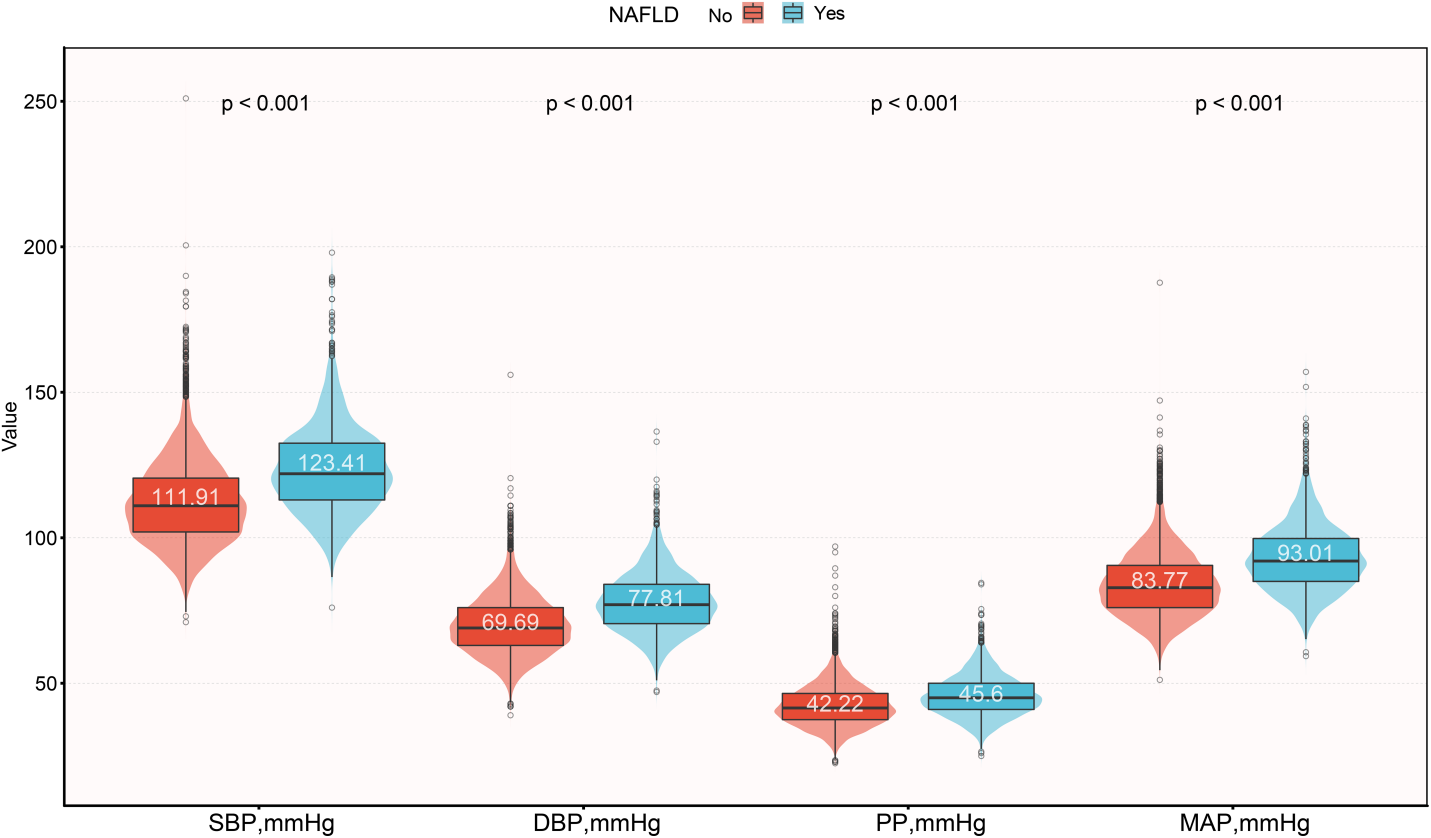
Violin plots show baseline characteristics of the four blood pressure indices in the NAFLD and non-NAFLD groups. NAFLD, non-alcoholic fatty liver disease; SBP, systolic blood pressure; DBP, diastolic blood pressure; PP, pulse pressure; MAP, mean arterial pressure.

Of the 14,251 participants included in this study, the male-to-female ratio was 1.08, with an average age of 43 years, and the prevalence of NAFLD was 17.59%. We compared the baseline characteristics of the study population grouped based on NAFLD diagnosis (Table 1), and observed significant differences (standardized difference >0.1) in all baseline characteristics except for drinking status and exercise habits. Compared to the non-NAFLD group, participants with NAFLD had relatively higher age, height, weight, BMI, WC, ALT, AST, GGT, TC, TG, FPG, HbA1c, and blood pressure indices. Among these, SBP, DBP, and MAP showed a larger difference (standardized difference value >0.8), while PP showed a relatively smaller difference (standardized difference value = 0.48) (Figure 2). In addition, it should also be noted that there was a large gap in the prevalence of NAFLD between the two groups [Women 478/6,840 (6.99%) vs. Men 2,029/7,411 (27.38%)], and the standardized difference value was further calculated to be 0.56.

**Table 1:** Characteristics of the study subjects with and without NAFLD.

	Non-NAFLD	NAFLD	Standardized difference
No of subjects	11,744	2,507	
Gender			0.78 (0.74, 0.83)
Women	6,362 (54.17%)	478 (19.07%)	
Men	5,382 (45.83%)	2,029 (80.93%)	
Age, years	42.00 (18.00–79.00)	44.00 (19.00–72.00)	0.18 (0.13, 0.22)
Weight, kg	57.72 (9.98)	72.18 (11.33)	1.35 (1.31, 1.40)
Height, cm	164.11 (8.44)	168.03 (7.90)	0.48 (0.44, 0.52)
BMI, kg/m^2^	21.33 (2.61)	25.50 (3.13)	1.45 (1.40, 1.49)
WC, cm	74.09 (7.92)	85.98 (7.79)	1.51 (1.47, 1.56)
ALT, IU/L	15.00 (2.00–856.00)	27.00 (6.00–220.00)	0.96 (0.91, 1.00)
AST, IU/L	17.00 (3.00–590.00)	20.00 (6.00–140.00)	0.56 (0.51, 0.60)
GGT, IU/L	14.00 (3.00–259.00)	23.00 (6.00–375.00)	0.61 (0.57, 0.66)
TC, mmol/L	5.06 (0.85)	5.44 (0.87)	0.45 (0.41, 0.49)
HDL-C, mmol/L	1.52 (0.40)	1.19 (0.29)	0.96 (0.92, 1.01)
TG, mmol/L	0.65 (0.07–10.27)	1.24 (0.16–7.69)	0.96 (0.91, 1.00)
FPG, mmol/L	5.09 (0.40)	5.39 (0.36)	0.78 (0.74, 0.82)
HbA1c, %	5.15 (0.31)	5.30 (0.33)	0.46 (0.42, 0.51)
SBP, mmHg	111.91 (14.02)	123.41 (14.83)	0.80 (0.75, 0.84)
DBP, mmHg	69.69 (9.85)	77.81 (10.19)	0.81 (0.77, 0.85)
PP, mmHg	42.22 ± 6.96	45.60 ± 7.13	0.48 (0.44, 0.52)
MAP, mmHg	83.77 ± 10.93	93.01 ± 11.46	0.83 (0.78, 0.87)
Exercise habits	2,093 (17.82%)	377 (15.04%)	0.08 (0.03, 0.12)
Drinking status			0.04 (−0.00, 0.09)
No or rarely	9,717 (82.74%)	2,088 (83.29%)	
Light	1,472 (12.53%)	286 (11.41%)	
Moderate	555 (4.73%)	133 (5.31%)	
Smoking status			0.35 (0.31, 0.39)
No	7,561 (64.38%)	1,185 (47.27%)	
Former	1,920 (16.35%)	639 (25.49%)	
Current	2,263 (19.27%)	683 (27.24%)	

NAFLD, non-alcoholic fatty liver disease; BMI, body mass index; WC, waist circumference; ALT, alanine aminotransferase; AST, aspartate aminotransferase; GGT, gamma-glutamyl transferase; HDL-C, high-density lipoprotein cholesterol; TC, total cholesterol; TG, triglyceride; HbA1c, hemoglobin A1c; FPG, fasting plasma glucose; SBP, systolic blood pressure; DBP, diastolic blood pressure; PP, pulse pressure; MAP, mean arterial pressure.

Values were expressed as mean (standard deviation) or median (interquartile range) or *n* (%).

### Assessment of the four blood pressure indices in estimating NAFLD risk

Table 2 presents the variations in the associations between the four blood pressure indices and NAFLD in progressively adjusted models. Initially, in the unadjusted model, all four indices were positively correlated with NAFLD; the corresponding OR values per SD increase for SBP, DBP, PP, and MAP were 2.16, 2.21, 1.58, 2.23, respectively, with MAP showing the largest OR value in relation to NAFLD. After adjusting for gender, age, height, and BMI in Model I, the association between all four indices and NAFLD substantially weakened, yet MAP still had the largest OR, while PP had the lowest. The results remained similar after further adjusting for lifestyle factors. Finally, to fully consider the impact of covariates, we adjusted for all non-collinear variables in Model III. The results indicated that compared to the other three blood pressure indices, MAP still had the strongest association with NAFLD (OR per SD increase: 1.20, 95% CI: 1.11–1.30). Additionally, it is worth noting that the association between PP and NAFLD disappeared in the fully adjusted model.

**Table 2:** Multivariable logistic regression analysis of the correlation between blood pressure index and NAFLD.

	OR (95% CI) (Per SD increase)			
	Unadjusted Model	Model I	Model II	Model III
SBP	2.16 (2.06, 2.26)	1.20 (1.13, 1.27)	1.22 (1.15, 1.29)	1.16 (1.08, 1.23)
DBP	2.21 (2.11, 2.31)	1.21 (1.14, 1.29)	1.23 (1.16, 1.31)	1.18 (1.11, 1.26)
PP	1.58 (1.51, 1.65)	1.10 (1.04, 1.16)	1.10 (1.05, 1.17)	0.97 (0.90, 1.04)
MAP	2.23 (2.12, 2.33)	1.22 (1.15, 1.29)	1.24 (1.16, 1.31)	1.20 (1.11, 1.30)

OR, Odds ratios; SD, standard deviation; other abbreviations as in Table 1.

Model I adjusted gender, age, height and BMI.

Model II adjusted model I + exercise habits, smoking status and smoking status.

Model III adjusted model II + ALT, AST, GGT, HDL-C, TC, TG, FPG and HbA1c.

Model III further adjust PP in the model with MAP as independent variable, and further adjust MAP in the model with PP as independent variable.

Considering that the prevalence of NAFLD was quite different between men and women in baseline characteristic analysis, it is necessary to further evaluate whether there is gender difference in the association between blood pressure indices and NAFLD. We further conducted stratified analyzes by gender and examined whether gender played a modifying role in the association using likelihood ratio tests. The results of these analyses revealed a significant positive correlation between blood pressure indices except PP and NAFLD in both sexes (Supplementary Table S5). In addition, blood pressure indices SBP, DBP, and MAP showed a stronger correlation with NAFLD in men compared to women, but further interactive tests showed that the difference was not statistically significant (All *P*-interactions >0.05).

### Sensitivity analysis

To verify the stability of the association between the four blood pressure indices and NAFLD, we conducted the same analysis in populations with relatively lower NAFLD risk. The results were consistent with the primary analysis across all subgroups, including those without exercise habits, aged <60 years, normotensive, and non-obese. The association between PP and NAFLD disappeared after full adjustment for confounders, while MAP showed a superior ability to assess NAFLD risk compared to SBP and DBP (Table 3).

**Table 3:** Adjusted odds ratios and 95% confidence intervals for NAFLD risk associated with the blood pressure index in different test populations: sensitivity analysis.

	OR (95% CI) (Per SD increase)			
	SBP	DBP	PP	MAP
Sensitivity-1	1.19 (1.11, 1.28)	1.20 (1.12, 1.29)	1.00 (0.93, 1.09)	1.20 (1.11, 1.30)
Sensitivity-2	1.19 (1.12, 1.27)	1.22 (1.14, 1.31)	0.96 (0.89, 1.03)	1.25 (1.15, 1.35)
Sensitivity-3	1.15 (1.05, 1.25)	1.18 (1.08, 1.28)	0.97 (0.90, 1.05)	1.20 (1.09, 1.32)
Sensitivity-4	1.35 (1.26, 1.46)	1.39 (1.29, 1.50)	0.98 (0.90, 1.06)	1.41 (1.28, 1.55)

OR, Odds ratios; SD, standard deviation; other abbreviations as in Table 1.

Models adjusted for the same covariates as in model III (Table 2).

(1) sensitivity-1: excluding subjects with exercise habits at baseline; (2) sensitivity-2: excluding subjects more than 60 years of age at baseline; (3) sensitivity-3: excluding subjects whose baseline SBP ≥ 140 mmHg or DBP ≥ 90 mmHg; (4) sensitivity-4: excluding subjects whose baseline BMI ≥ 25 kg/m^2^.

Exercise habits was not included in model III of sensitivity-1; age was not included in model III of sensitivity-2; BMI was not included in model III of sensitivity-4.

### Assessment of the four blood pressure indices in identifying NAFLD

**Figure 3:**
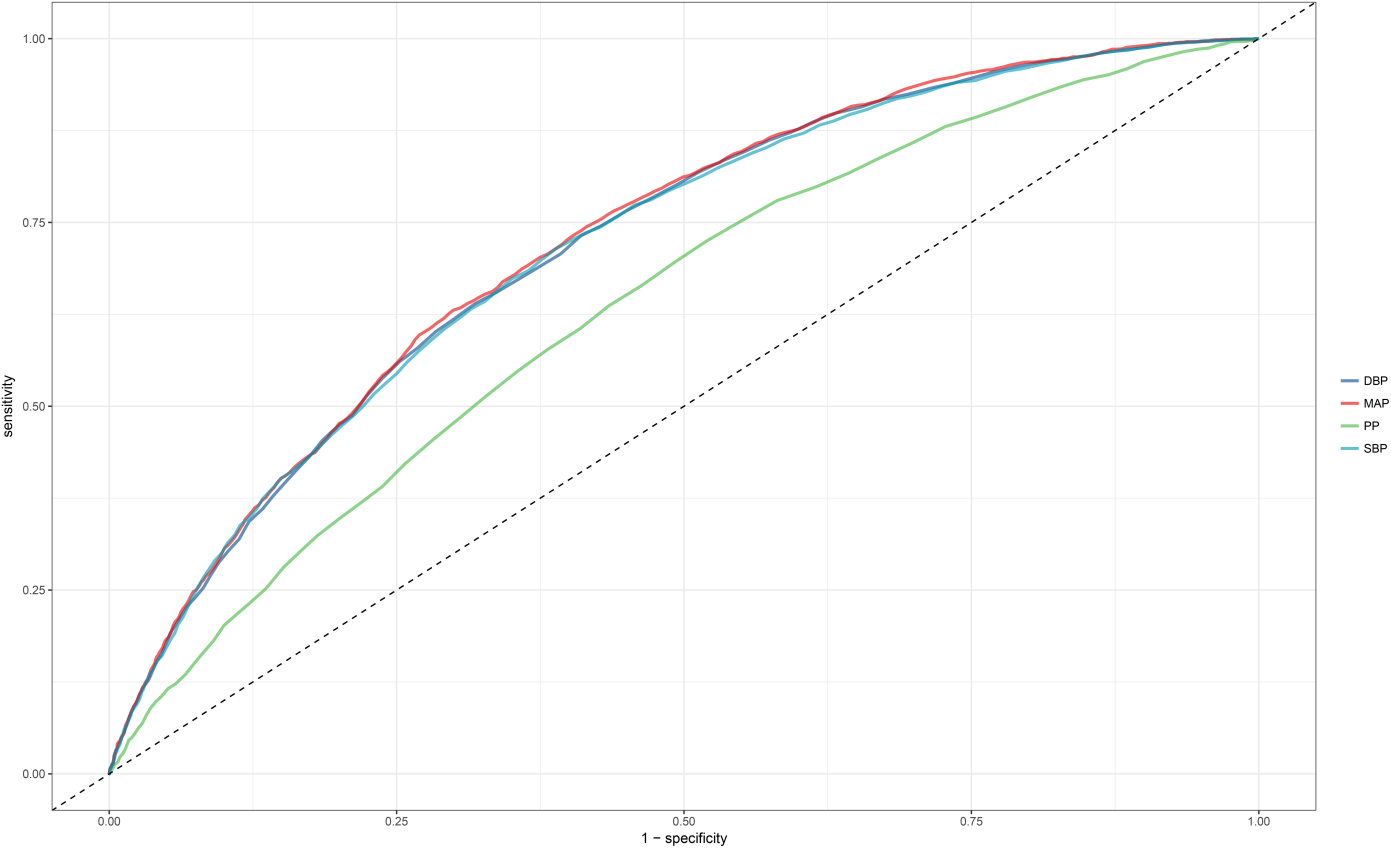
Area under the receiver operating characteristic curve for SBP, DBP, PP, and MAP for identification of NAFLD in the entire population. NAFLD, non-alcoholic fatty liver disease; SBP, systolic blood pressure; DBP, diastolic blood pressure; PP, pulse pressure; MAP, mean arterial pressure.

ROC curves were plotted to evaluate the ability of the four blood pressure indices to identify NAFLD (Figure 3). The results indicated that MAP had the highest AUC value (0.7258), followed by DBP (0.7210), SBP (0.7196), and finally PP (0.6379) (Table 4). After further comparison using the Delong test, significant statistical differences were found between MAP and SBP, DBP, and PP in identifying NAFLD (All Delong *P *< 0.05).

**Table 4:** The best threshold, sensitivities, specificities, and area under the curve of blood pressure index for the screening of NAFLD in the general population.

	AUC	95% CI low	95% CI upp	Best threshold	Specificity	Sensitivity
SBP*	0.7196	0.7090	0.7301	114.7500	0.6116	0.7140
DBP*	0.7210	0.7106	0.7315	71.2500	0.5897	0.7320
PP*	0.6379	0.6263	0.6494	41.2500	0.4799	0.7252
MAP	0.7258	0.7154	0.7361	88.7500	0.7008	0.6306

AUC, area under the curve; CI, confidence interval; Other abbreviations as in Table 1.

*P < 0.05 compared with MAP.

## Discussion

In this survey analysis based on a health examination population, after adequately adjusting for confounding factors, we found that all blood pressure indices, except PP, were positively correlated with NAFLD. Among these, MAP may be the optimal index for assessing NAFLD risk. Additionally, in terms of identifying NAFLD, MAP was the most accurate compared to SBP, DBP, and PP. These findings emphasized that MAP might be the most promising blood pressure index in screening for NAFLD.

The association between blood pressure indices and NAFLD has been reported in many studies, with published evidence generally supporting a positive correlation between SBP, DBP, PP, MAP, and NAFLD (14–16, 31–34). However, it's noteworthy that most of these studies focused on evaluating the association of individual blood pressure indices with NAFLD and rarely assessed the impact of different indices on NAFLD risk simultaneously. To our knowledge, only one study has investigated the association between multiple blood pressure indices and fatty liver; Patel et al.'s study based on a British birth cohort observed a positive correlation between fatty liver and SBP, DBP, and MAP in the same adolescent cohort, while no significant correlation with PP (35). The findings about PP in Patel et al.'s study are inconsistent with previous results from Zhang et al. based on Mendelian randomization (15), possibly due to the lack of adjustment for confounders in Zhang et al.'s study, masking the true association between PP and NAFLD. In our current study, a positive correlation between PP and NAFLD was observed in the unadjusted and partially adjusted models (Models I and II), but this association disappeared in the fully adjusted model (Model III). Therefore, combining the results of our study with Patel et al.'s (35), we believe that PP has a weak and unstable, non-independent positive correlation with NAFLD. Furthermore, considering the significant differences in magnitude among blood pressure indices, we performed Z-transformation and calculated the OR values of all blood pressure indices per SD increase in relation to NAFLD. Compared to SBP and DBP, MAP had the highest degree of association with NAFLD. This finding was further confirmed in several sensitivity analyses (Table 3); in our study, among the four indices, MAP had the strongest association with NAFLD in populations without exercise habits, non-obese, relatively younger, and normotensive, while the association between PP and NAFLD disappeared after full adjustment for confounders. Compared to Patel et al.'s study (35), our study population consisted of adults, with a larger sample size (14,251 vs. 1,904), and data normalization was conducted for relative comparability of effect sizes. Based on the findings of the association analysis in our current study, MAP might be the best blood pressure index for assessing NAFLD risk.

The gender difference of NAFLD has been widely concerned in recent years. Generally speaking, the prevalence of NAFLD in men is higher than that in women (36–38), and the epidemiological survey based on the Japanese population shows that the prevalence of NAFLD in men is about three times that of women (39, 40), which is highly similar to the results of current research. Compared with women, men usually show more visceral fat deposition, are more susceptible to leptin resistance, lack estrogen receptors, and tend to synthesize fatty acids into fat storage (38). Considering the significant gender difference in the prevalence of NAFLD, in the current study, we further evaluated whether the association between blood pressure indices and NAFLD was modified by gender, and the results were consistent with the main analysis: In both sexes, except for PP, the correlation between blood pressure indices and NAFLD is significant, and the correlation between MAP and NAFLD is the highest in both sexes. However, in further interactive tests, we did not detect a significant gender difference in the correlation between blood pressure indices and NAFLD.

Reports on blood pressure indices in identifying NAFLD are currently quite limited. Most published studies have primarily focused on revealing the association between blood pressure indices and NAFLD (14–16, 31–34), affirming the importance of blood pressure indices in NAFLD risk assessment, even at normal blood pressure levels. After establishing this association, further evaluating the value of blood pressure indices in identifying NAFLD is worth researching and emphasizing. In a recent study by Xu et al., they assessed the value of SBP, DBP, and MAP in identifying NAFLD in a non-obese population, noting that MAP had the highest value in identifying NAFLD compared to SBP and DBP (34). Similarly to Xu et al.'s findings, our study, including both obese and non-obese individuals in a general health examination population, found MAP to have the highest value in identifying NAFLD (AUC: 0.7258). Combining the results of the ROC analysis and the standardized OR values from the association analysis, MAP seems to be the most useful blood pressure index for screening NAFLD in a health examination population.

After establishing that MAP was the most advantageous blood pressure index for screening NAFLD, further estimating its effective clinical threshold is valuable. It's noteworthy that MAP has only recently gained popularity in epidemiological studies, previously being primarily used in critical and perioperative clinical monitoring, due to its importance in reflecting vital organ perfusion (41–43). Although there are no official recommendations for the optimal MAP target in critically ill or perioperative patients, literature reviews suggest that maintaining MAP above 65 mmHg is crucial for patient recovery (44–46). For survivors of myocardial infarction, studies showed that maintaining MAP above 80 was important for improving adverse outcomes (47). Additionally, recent epidemiological surveys have proposed gender-specific MAP thresholds for predicting metabolic syndrome in the elderly at 84 mmHg (male) and 83.3 mmHg (female) (48), a MAP threshold of 92.833 mmHg for predicting diabetes (49), and 88 mmHg (male) and 89 mmHg (female) for predicting non-obese NAFLD (34). In our current study, based on general health examination population data, we found the threshold for identifying NAFLD using MAP to be 88.75 mmHg. Based on these results, we suggest that a MAP threshold of 90 mmHg might be appropriate for assessing various chronic diseases, including NAFLD.

Reflections on the clinical significance of the relationship between blood pressure indices and NAFLD can provide insights for subsequent research and clinical applications. Similar to some past studies investigating the relationship between multiple blood pressure indices and diseases (48, 50–59), our study is the first to identify MAP as the most promising blood pressure index for screening NAFLD. These findings are significant because they (i) fill a knowledge gap in NAFLD; (ii) provide ideas and important references for subsequent clinical studies or mechanistic research related to NAFLD; (iii) offer reliable data and insights for future NAFLD risk modeling studies or model improvement research; (iv) considering the high prevalence of NAFLD, the most important clinical significance of this study lies in its application to NAFLD screening, as blood pressure indices are easily obtainable and self-measurable, making them valuable for healthcare professionals and individuals for daily NAFLD identification/risk assessment.

Our study has some limitations that need to be addressed: (i) NAFLD diagnosis in our study was based on ultrasonography, which might miss some cases with mild hepatic steatosis (60). (ii) The cross-sectional design limits the study's ability to further explore the predictive value of blood pressure indices for NAFLD. (iii) The evidence from our study is applicable to the Japanese population and needs further validation in other populations. (iv) Although we have made comprehensive adjustments with the available data, some unmeasured factors were not included in our analysis, which could lead to some residual confounding (61). (v) Since June 2023, it has gradually become noteworthy that the term NAFLD is being replaced by the new term “Metabolic dysfunction-associated steatotic liver disease (MASLD),” as MASLD is considered to better align with the pathophysiological characteristics of the disease (62–64). Furthermore, the definition of MASLD further emphasizes the significant impact of cardiac metabolic factors on disease diagnosis (62). Given that blood pressure index is one of the most crucial cardiac metabolic factors (56, 65, 66), and multiple blood pressure indices have been established in current research for screening NAFLD, we speculate that blood pressure indices may similarly play a significant role in MASLD screening; however, further research is still needed.

## Conclusion

We discovered that, in a general population, SBP, DBP, and MAP all positively correlate with NAFLD, except for PP. After data normalization, MAP showed the strongest association with NAFLD. Furthermore, subsequent ROC analysis revealed that MAP was the most accurate blood pressure index for identifying NAFLD compared to SBP, DBP, and PP. These findings highlight a key point: MAP may be the most important blood pressure index for assessing NAFLD.

## Data Availability

The original contributions presented in the study are included in the article/Supplementary Materials, further inquiries can be directed to the corresponding authors.
