# Supplementary material for: Evaluating the relative importance of different blood pressure indices in screening for NAFLD: a survey report based on a health examination population
Source: Front Cardiovasc Med. 2024 Apr 29;11:1338156. doi: 10.3389/fcvm.2024.1338156 (PMC11089114; doi:10.3389/fcvm.2024.1338156)
Supplement: Supplementary file 1 [file Table1.docx]

Supplementary Table 1: Collinearity diagnostics steps of SBP with other covariates.

|  | VIF | | | | | |
| --- | --- | --- | --- | --- | --- | --- |
|  | Step 1 | Step 2 | Step 3 | Step 4 | Step 5 | Step 6 |
| SBP, mmHg | Inf | Inf | 15.1 | 15.1 | 1.4 | 1.4 |
| Sex | 3.3 | 3.3 | 3.3 | 3.3 | 3.3 | 3.2 |
| Age, years | 1.4 | 1.4 | 1.4 | 1.4 | 1.4 | 1.3 |
| Weight, kg | 168.5 | 168.5 | 168.5 | NA | NA | NA |
| Height, cm | 51.9 | 51.9 | 51.9 | 2.9 | 2.9 | 2.4 |
| BMI, kg/m^2^ | 95.9 | 95.9 | 95.9 | 5 | 5 | 1.7 |
| WC, cm | 5.9 | 5.9 | 5.9 | 5.9 | 5.9 | NA |
| ALT, IU/L | 4.1 | 4.1 | 4.1 | 4.1 | 4.1 | 4.1 |
| AST, IU/L | 3.3 | 3.3 | 3.3 | 3.3 | 3.3 | 3.3 |
| GGT, IU/L | 1.5 | 1.5 | 1.5 | 1.5 | 1.5 | 1.5 |
| HDL-C, mml/L | 1.8 | 1.8 | 1.8 | 1.8 | 1.8 | 1.8 |
| TC, mmol/L | 1.5 | 1.5 | 1.5 | 1.5 | 1.5 | 1.5 |
| TG, mmol/L | 1.7 | 1.7 | 1.7 | 1.7 | 1.7 | 1.7 |
| FPG, mmol/L | 1.5 | 1.5 | 1.5 | 1.5 | 1.5 | 1.5 |
| HbA1c, % | 1.2 | 1.2 | 1.2 | 1.2 | 1.2 | 1.2 |
| Exercise habits | 1 | 1 | 1 | 1 | 1 | 1 |
| Drinking status | 1.2 | 1.2 | 1.2 | 1.2 | 1.2 | 1.2 |
| Smoking status | 1.4 | 1.4 | 1.4 | 1.4 | 1.4 | 1.4 |
| DBP, mmHg | Inf | NA | NA | NA | NA | NA |
| PP, mmHg | Inf | Inf | NA | NA | NA | NA |
| MAP, mmHg | Inf | Inf | 15.6 | 15.5 | NA | NA |

Abbreviations: Inf: infinity; VIF: Variance inflation factor; Other abbreviations as in Table ​1.

Note-1: Variance inflation factor = 1/(1-R^2^). Abbreviations as in Table 1.

Note-2: The variables with Variance inflation factor >5 will be regarded as collinear variables and cannot be included in the multiple regression model.

Supplementary Table 2: Collinearity diagnostics steps of DBP with other covariates.

|  | VIF | | | | | |
| --- | --- | --- | --- | --- | --- | --- |
|  | Step 1 | Step 2 | Step 3 | Step 4 | Step 5 | Step 6 |
| DBP, mmHg | Inf | Inf | 29.7 | 29.7 | 1.4 | 1.4 |
| Sex | 3.3 | 3.3 | 3.3 | 3.3 | 3.3 | 3.3 |
| Age, years | 1.4 | 1.4 | 1.4 | 1.4 | 1.4 | 1.3 |
| Weight, kg | 168.5 | 168.5 | 168.5 | NA | NA | NA |
| Height, cm | 51.9 | 51.9 | 51.9 | 2.9 | 2.9 | 2.4 |
| BMI, kg/m^2^ | 95.9 | 95.9 | 95.9 | 5 | 5 | 1.7 |
| WC, cm | 5.9 | 5.9 | 5.9 | 5.9 | 5.9 | NA |
| ALT, IU/L | 4.1 | 4.1 | 4.1 | 4.1 | 4.1 | 4.1 |
| AST, IU/L | 3.3 | 3.3 | 3.3 | 3.3 | 3.3 | 3.3 |
| GGT, IU/L | 1.5 | 1.5 | 1.5 | 1.5 | 1.5 | 1.5 |
| HDL-C, mmol/L | 1.8 | 1.8 | 1.8 | 1.8 | 1.8 | 1.8 |
| TC, mmol/L | 1.5 | 1.5 | 1.5 | 1.5 | 1.5 | 1.5 |
| TG, mmol/L | 1.7 | 1.7 | 1.7 | 1.7 | 1.7 | 1.7 |
| FPG, mmol/L | 1.5 | 1.5 | 1.5 | 1.5 | 1.5 | 1.5 |
| HbA1c, % | 1.2 | 1.2 | 1.2 | 1.2 | 1.2 | 1.2 |
| Exercise habits | 1 | 1 | 1 | 1 | 1 | 1 |
| Drinking status | 1.2 | 1.2 | 1.2 | 1.2 | 1.2 | 1.2 |
| Smoking status | 1.4 | 1.4 | 1.4 | 1.4 | 1.4 | 1.4 |
| SBP, mmHg | Inf | NA | NA | NA | NA | NA |
| PP, mmHg | Inf | Inf | NA | NA | NA | NA |
| MAP, mmHg | Inf | Inf | 30.2 | 30.1 | NA | NA |

Abbreviations: Inf: infinity; VIF: Variance inflation factor; Other abbreviations as in Table ​1.

Note-1: Variance inflation factor = 1/(1-R^2^). Abbreviations as in Table 1.

Note-2: The variables with Variance inflation factor >5 will be regarded as collinear variables and cannot be included in the multiple regression model.

Supplementary Table 1: Collinearity diagnostics steps of MAP with other covariates.

|  | VIF | | | | |
| --- | --- | --- | --- | --- | --- |
|  | Step 1 | Step 2 | Step 3 | Step 4 | Step 5 |
| MAP, mmHg | Inf | Inf | 2 | 2 | 2 |
| Sex | 3.3 | 3.3 | 3.3 | 3.3 | 3.3 |
| Age, years | 1.4 | 1.4 | 1.4 | 1.4 | 1.3 |
| Weight, kg | 168.5 | 168.5 | 168.5 | NA | NA |
| Height, cm | 51.9 | 51.9 | 51.9 | 2.9 | 2.4 |
| BMI, kg/m^2^ | 95.9 | 95.9 | 95.9 | 5 | 1.7 |
| WC, cm | 5.9 | 5.9 | 5.9 | 5.9 | NA |
| ALT, IU/L | 4.1 | 4.1 | 4.1 | 4.1 | 4.1 |
| AST, IU/L | 3.3 | 3.3 | 3.3 | 3.3 | 3.3 |
| GGT, IU/L | 1.5 | 1.5 | 1.5 | 1.5 | 1.5 |
| HDL-C, mmol/L | 1.8 | 1.8 | 1.8 | 1.8 | 1.8 |
| TC, mmol/L | 1.5 | 1.5 | 1.5 | 1.5 | 1.5 |
| TG, mmol/L | 1.7 | 1.7 | 1.7 | 1.7 | 1.7 |
| FPG, mmol/L | 1.5 | 1.5 | 1.5 | 1.5 | 1.5 |
| HbA1c, % | 1.2 | 1.2 | 1.2 | 1.2 | 1.2 |
| Exercise habits | 1 | 1 | 1 | 1 | 1 |
| Drinking status | 1.2 | 1.2 | 1.2 | 1.2 | 1.2 |
| Smoking status | 1.4 | 1.4 | 1.4 | 1.4 | 1.4 |
| SBP, mmHg | Inf | NA | NA | NA | NA |
| DBP, mmHg | Inf | Inf | NA | NA | NA |
| PP, mmHg | Inf | Inf | 1.5 | 1.5 | 1.5 |

Abbreviations: Inf: infinity; VIF: Variance inflation factor; Other abbreviations as in Table ​1.

Note-1: Variance inflation factor = 1/(1-R^2^). Abbreviations as in Table 1.

Note-2: The variables with Variance inflation factor >5 will be regarded as collinear variables and cannot be included in the multiple regression model.

Supplementary Table 4: Collinearity diagnostics steps of PP with other covariates.

|  | VIF | | | | |
| --- | --- | --- | --- | --- | --- |
|  | Step 1 | Step 2 | Step 3 | Step 4 | Step 5 |
| PP, mmHg | Inf | Inf | 1.3 | 1.3 | 1.3 |
| Sex | 3.3 | 3.3 | 3.3 | 3.3 | 3.3 |
| Age, years | 1.4 | 1.4 | 1.4 | 1.4 | 1.3 |
| Weight, kg | 168.5 | 168.5 | 168.5 | NA | NA |
| Height, cm | 51.9 | 51.9 | 51.9 | 2.9 | 2.4 |
| BMI, kg/m^2^ | 95.9 | 95.9 | 95.9 | 5 | 1.7 |
| WC, cm | 5.9 | 5.9 | 5.9 | 5.9 | NA |
| ALT, IU/L | 4.1 | 4.1 | 4.1 | 4.1 | 4.1 |
| AST, IU/L | 3.3 | 3.3 | 3.3 | 3.3 | 3.3 |
| GGT, IU/L | 1.5 | 1.5 | 1.5 | 1.5 | 1.5 |
| HDL-C, mmol/L | 1.8 | 1.8 | 1.8 | 1.8 | 1.8 |
| TC, mmol/L | 1.5 | 1.5 | 1.5 | 1.5 | 1.5 |
| TG, mmol/L | 1.7 | 1.7 | 1.7 | 1.7 | 1.7 |
| FPG, mmol/L | 1.5 | 1.5 | 1.5 | 1.5 | 1.5 |
| HbA1c, % | 1.2 | 1.2 | 1.2 | 1.2 | 1.2 |
| Exercise habits | 1 | 1 | 1 | 1 | 1 |
| Drinking status | 1.2 | 1.2 | 1.2 | 1.2 | 1.2 |
| Smoking status | 1.4 | 1.4 | 1.4 | 1.4 | 1.4 |
| SBP, mmHg | Inf | NA | NA | NA | NA |
| MAP, mmHg | Inf | Inf | NA | NA | NA |
| DBP, mmHg | Inf | Inf | 1.6 | 1.6 | 1.6 |

Abbreviations: Inf: infinity; VIF: Variance inflation factor; Other abbreviations as in Table ​1.

Note-1: Variance inflation factor = 1/(1-R^2^). Abbreviations as in Table 1.

Note-2: The variables with Variance inflation factor >5 will be regarded as collinear variables and cannot be included in the multiple regression model.

Supplementary Table 5: Evaluating the association between blood pressure indices and NAFLD according to gender stratification.

|  | OR (95% CI) (Per SD increase) | | *P*-interaction |
| --- | --- | --- | --- |
|  | Men | Women |  |
| SBP | 1.18 (1.09, 1.27) | 1.11 (1.00, 1.23) | 0.3269 |
| DBP | 1.18 (1.10, 1.28) | 1.17 (1.05, 1.32) | 0.8970 |
| PP | 1.00 (0.92, 1.09) | 0.90 (0.81, 1.01) | 0.1083 |
| MAP | 1.21 (1.11, 1.32) | 1.17 (1.04, 1.33) | 0.6372 |

Abbreviations: OR: Odds ratios; SD: standard deviation; other abbreviations as in Table ​1.

Model adjusted age, height and BMI, exercise habits, drinking status, smoking status, ALT, AST, GGT, HDL-C, TC, TG, FPG and HbA1c.

Note: In the model with PP as the independent variable, MAP was further adjusted; In the model with MAP as the independent variable, PP was further adjusted.
